# Antenatal corticosteroids and outcomes into adulthood

**DOI:** 10.1111/ppe.12919

**Published:** 2022-08-10

**Authors:** Lex W. Doyle

**Affiliations:** ^1^ Neonatal Services Royal Women's Hospital Melbourne Victoria Australia; ^2^ Clinical Sciences Murdoch Children's Research Institute Melbourne Victoria Australia; ^3^ Department of Obstetrics and Gynaecology University of Melbourne Melbourne Victoria Australia; ^4^ Department of Paediatrics University of Melbourne Melbourne Victoria Australia

It is now 50 years since the first report from New Zealand of a randomised controlled trial (RCT) of antenatal corticosteroids (ACS) to accelerate fetal lung maturation in humans.^1^ This RCT had been prompted by the observation that ACS given to sheep resulted in lambs born preterm having less respiratory distress after birth than was expected. Many RCTs in humans followed the initial report by Liggins and Howie,^1^ the results of which have been synthesised in several Cochrane reviews, the most recent of which have been updated in 2020^2^ and 2022.^3^ The cumulative evidence is that ACS are beneficial for pregnancies ≤35 weeks' gestation, with reductions in neonatal death (risk ratio [RR] 0.77, 95% confidence interval [CI] 0.69, 0.86; 19 studies, 6961 infants), and major morbidities including respiratory distress syndrome (RDS) (RR 0.70, 95% CI 0.63, 0.78; 20 studies, 7041 infants) and any intraventricular haemorrhage (IVH) (RR 0.56, 95% CI 0.44, 0.72; 11 studies, 5412 infants).^2^ If the risk of preterm delivery persists after 1 week, there is evidence that repeated courses of ACS reduce RDS (RR 0.82, 95% CI 0.74, 0.90; 9 studies, 3540 infants), but with little evidence for an effect on neonatal death (RR 0.91, 95% CI 0.62, 1.34; 7 studies, 2758 infants) or any IVH (RR 0.95, 95% CI 0.75, 1.19; 6 studies 3223 infants).^3^

Although the short‐term outcomes are favourable, there are far less data about outcomes into school‐age, and even less into adulthood. Long‐term health for the fetus could be affected through programming in utero, so short‐term benefits might be outweighed by adverse longer term effects. The limited data from RCTs that do exist suggest no beneficial or adverse effects, with few exceptions. For example, in the Cochrane review by McGoldrick et al.,^2^ developmental delay in childhood was reduced in the ACS group (RR 0.51, 95% CI 0.27, 0.97; 3 studies, 600 children), but not intellectual impairment in childhood (RR 0.86, 95% CI 0.44, 1.69; 3 studies, 778 children). IQ and educational achievement at 31 years of age were similar in 87 adults exposed to ACS and 105 in the control group from the original Liggins study.^4^ However, from the same study there was some evidence suggesting insulin resistance at 30 years in those exposed to ACS.^5^

Doubts about possible adverse long‐term outcomes will remain without conclusive evidence to the contrary, which is currently not available from the reported RCTs. Since many of the RCTS have been completed a long time ago, participants involved in the original RCTs are unlikely to be tracked into adulthood in the future, to try to fill the gaps in knowledge.

In the absence of data from RCTs, the field becomes more reliant on observational data. In the current issue of the journal, Darlow et al. have reported the outcomes of adults born with a birth weight <1500 g in New Zealand in 1986.^6^ The cohort was originally selected to study the long‐term effects of retinopathy of prematurity. At 26–30 years of age there was little evidence for any associations of ACS exposure with any cardiovascular, metabolic, respiratory, or cognitive outcomes. There was a suggestion of an increase in depression in those exposed to ACS, but the authors concluded that this finding required further corroboration. That only 58% of the 250 participants assessed had been exposed to ACS in 1986 reflects, in part, how slowly ACS have been translated into clinical practice, even in New Zealand.

There are several problems in relying on observational data to weigh the balance between the three possible mutually exclusive outcomes for a fetus following exposure to ACS: (1) death, (2) survival with adverse long‐term outcomes or (3) survival free of adverse long‐term outcomes. Firstly, stillbirths are excluded in studies that include only live births, which apply to most extant observational cohorts. Secondly, there could be a bias towards better survival for those who received ACS, particularly for those born extremely preterm (EP; <28 weeks' gestation). ACS improve survival, but their use may also reflect an attitude of offering more active care, including transferring the pregnant mother to birth in a tertiary medical centre with immediate access to neonatal intensive care for the infant after birth. This is relevant as EP live births in tertiary medical centres have considerably higher survival rates than do those born EP outside such centres,^7^ and infants <29 weeks' gestation who are inborn have better long‐term outcomes than those who are outborn.^8^ Possibly counteracting any increase in survival is that more immature infants who survive have higher rates of adverse health outcomes than those who are more mature. Adjustment for known confounding variables, such as place of birth, can only remove some of the bias between those exposed and those unexposed to ACS; one cannot adjust for unknown confounders. Thirdly, cohorts selected with upper birth weight or gestational age limits do not obtain all pregnancy outcomes where the fetus has been exposed to ACS because many women ultimately deliver larger babies or much later, even at term, with no immediate complications for the infant. Under these circumstances, the fetus is exposed only to the potential disadvantages of ACS and not to any of the benefits.

A possible solution would be to obtain data from complete geographical cohorts with antenatal care and pregnancy outcomes, including stillbirth, linked to long‐term health outcomes for the offspring. For example, some Scandinavian countries are able to link data through the life course, and could potentially examine the association between adult outcomes and ACS exposure in huge whole‐population data sets. Even if adverse long‐term health issues are suspected through such linkage studies, this would not mean that ACS should not be used in pregnancies with threatened birth <35 weeks' gestation.^9^ It would be a question of weighing up the known survival advantages against any possible long‐term health harms, and those harms would have to be substantial to counteract the clear survival advantages. So far, the limited data into adulthood, including in the current study by Darlow et al.,^6^ have not unearthed any major disadvantages. More observational studies are required over the life course before we will as sure as we can be that the short‐term advantages of ACS are not outweighed by any long‐term disadvantages.

Due to the established benefits of ACS in relation to survival and major neonatal morbidities, future placebo‐controlled RCTs of ACS in pregnancies <35 weeks' gestation would be considered unethical, though RCTs comparing different doses of corticosteroids and different types of corticosteroids would not be unethical, and are needed. In contrast, in more mature pregnancies there is plenty of scope for placebo‐controlled RCTs at term or late term, and in pregnancies complicated by diabetes, which has been an exclusion for most of the extant RCTs of ACS. Given that almost all infants born late preterm or at term will survive, a benefit of ACS on mortality is unrealistic and hence it will be vital to follow survivors from RCTs in more mature groups well into childhood, and ideally into adulthood, to establish if there are any adverse sequelae that are not known about today.

Individuals who have been exposed to ACS in utero cannot do anything about their exposure many years later. However, they may want to know if such exposure increases their risks for any adverse outcomes, some of which may be modifiable (e.g., elevated blood pressure^10^). Observational studies such as those of Darlow et al. provide some reassurance about an absence of adverse outcomes, but need to be supported by more studies and larger numbers of participants exposed to ACS with outcomes into adulthood.

## About the author

**Lex W. Doyle** is Professor of Neonatal Paediatrics in the Department of Obstetrics and Gynecology at the University of Melbourne, and Associate Director of Research at the Royal Women's Hospital, Melbourne. His research over many years has focussed on improving the long‐term health outcomes of our most vulnerable newborn infants; those born extremely preterm (<28 weeks' gestation) or extremely low birth weight (<1000 g).

## AUTHOR CONTRIBUTIONS

Lex W Doyle conceived, wrote and approved the submitted version of this article.

## CONFLICT OF INTEREST

The author has no conflicts to declare.

## Data Availability

Data sharing is not applicable to this article as no new data were created or analyzed in this study.
